# Modeling Dynamic Introduction of Chikungunya Virus in the United States

**DOI:** 10.1371/journal.pntd.0001918

**Published:** 2012-11-29

**Authors:** Diego Ruiz-Moreno, Irma Sanchez Vargas, Ken E. Olson, Laura C. Harrington

**Affiliations:** 1 Department of Ecology and Evolutionary Biology, Cornell University, Ithaca, New York, United States of America; 2 Arthropod-Borne and Infectious Diseases Laboratory, Department of Microbiology, Immunology and Pathology, Colorado State University, Fort Collins, Colorado, United States of America; 3 Department of Entomology, Cornell University, Ithaca, New York, United States of America; Yale University, United States of America

## Abstract

Chikungunya is a mosquito-borne viral infection of humans that previously was confined to regions in central Africa. However, during this century, the virus has shown surprising potential for geographic expansion as it invaded other countries including more temperate regions. With no vaccine and no specific treatment, the main control strategy for Chikungunya remains preventive control of mosquito populations. In consideration for the risk of Chikungunya introduction to the US, we developed a model for disease introduction based on virus introduction by one individual. Our study combines a climate-based mosquito population dynamics stochastic model with an epidemiological model to identify temporal windows that have epidemic risk. We ran this model with temperature data from different locations to study the geographic sensitivity of epidemic potential. We found that in locations with marked seasonal variation in temperature there also was a season of epidemic risk matching the period of the year in which mosquito populations survive and grow. In these locations controlling mosquito population sizes might be an efficient strategy. But, in other locations where the temperature supports mosquito development all year the epidemic risk is high and (practically) constant. In these locations, mosquito population control alone might not be an efficient disease control strategy and other approaches should be implemented to complement it. Our results strongly suggest that, in the event of an introduction and establishment of Chikungunya in the US, endemic and epidemic regions would emerge initially, primarily defined by environmental factors controlling annual mosquito population cycles. These regions should be identified to plan different intervention measures. In addition, reducing vector: human ratios can lower the probability and magnitude of outbreaks for regions with strong seasonal temperature patterns. This is the first model to consider Chikungunya risk in the US and can be applied to other vector borne diseases.

## Author Summary

Chikungunya fever is a mosquito-borne viral infection showing a surprising potential for geographic expansion. Similar to other tropical infectious diseases having no vaccine and no specific treatment, the main control strategy for Chikungunya remains reduction of mosquito population size. We developed a model for disease introduction that combines a climate based mosquito population dynamics stochastic model with an epidemiological model in order to identify temporal windows during which disease introduction through one exposed individual might compromise the health status of the entire human population. We ran this model with temperature data from different locations showing the geographic sensitivity of this risk. The identification of temporal windows with epidemic risk at different spatial locations is key to guiding mosquito population control campaigns. Locations with marked seasonal variation also have a season with high epidemic risk matching the period in which mosquito populations survive and grow, therefore controlling mosquito population sizes might be an optimal strategy in those areas. However, locations with other temperature patterns may need additional control strategies to avoid epidemics. To our knowledge, this is the first model to explore Chikungunya introduction in the USA. Our modeling approach can be used for other vector borne diseases and can be expanded to compare the outcome with different control strategies.

## Introduction

Chikungunya fever (CHIKF) is a mosquito-borne viral infection first isolated in Tanzania in 1953 [1], [2]. CHIKF is caused by Chikungunya virus (CHIKV), an alphavirus with different variants endemic to countries in Africa and Southeast Asia [3], [4]. Illness caused by CHIKV is usually diagnosed based on symptoms, and often confused with dengue given some overlapping symptomology [5]. One symptom specific for Chikungunya is a debilitating and prolonged joint pain, affecting the peripheral small joints [6], that appears in conjunction with other nonspecific symptoms including fever, severe joint pain, muscle pain, headache, nausea, fatigue and occasionally rash [7]–[11]. CHIKF-related mortality is rare, but can occur, often in patients with other health conditions [1], [9], [11], [12]. There is no specific treatment for the disease; consequently, treatment is focused on symptomatic care and mosquito vector control. No vaccines are currently available for prevention of CHIKV infection, although vaccine candidates currently are under investigation [13].

The onset of the symptoms occurs after an intrinsic incubation period in the human host of approximately 4 days post infection [1], [8]–[11], and viremia in infective individuals usually persists for a period of approximately 7 days [1], [3], [14]. During this period, mosquitoes may be infected with CHIKV when feeding on viremic hosts. After the acute stage of infection, severe joint pain may persist for long periods in affected individuals. Some people show mild to no overt signs of illness. Seroprevalence studies have demonstrated that 25% of infected individuals have mild symptoms or were asymptomatic [3].

Laboratory studies have demonstrated that CHIKV disseminates to the salivary glands in competent mosquitoes quickly, within 2 days (range 1–14 days) post-infection [15]. Once infectious, mosquito vectors are thought to remain infectious for their lifetime. Prior to 2000, *Aedes aegypti* was the most important vector of CHIKV [6] , with *Ae. albopictus* considered a secondary vector [16]. Within the last decade several epidemics of CHIKF were reported. In 2005–2006, a severe epidemic occurred in Réunion Island [3], followed shortly after by epidemics in India [3], Southeast Asia [17] and other Indian Ocean islands [1], [18]. Sampling during the Reunion Island epidemic provided evidence for the role of *Ae. albopictus* as the main vector [19]–[21]. Sequencing of the envelope protein of the Reunion Island CHIKV isolates (CHIKV 226OPY1) showed that the outbreak was caused by a new variant of the virus with a single adaptive mutation. This single amino acid change from Alanine to Valine in the E1 glycoprotein at position 226 [22] increased infection and dissemination in *Ae. albopictus* [23]. Another epidemic of CHIKV OPY1 genotype occurred in Italy in 2007 [24], [25]. Epidemiological studies strongly implicate introduction of the virus from India by a traveler [21], [26]. This unexpected outbreak is a striking example of disease introduction in an area recently colonized by *Ae. albopictus* [15], [21], [27]. Moreover, it highlights the fact that CHIKV outbreaks can originate from just one infective individual even in temperate areas with seasonal transmission of arboviruses [25], [19].

The Asian tiger mosquito, *Ae. albopictus*, is an invasive urban mosquito native to East Asia [28]. It is a diurnally active species and thought to have a broader host range than *Ae. aegypti*, although in some regions it can be highly anthropophagic when human hosts are readily available [29]. In the past couple of decades this species has invaded many countries through the transport of goods, especially used tires, and increasing international travel [30]–[32]. Native to tropical regions of Asia, *Ae. albopictus* has successfully adapted to cooler climates within the 10°C isotherm [33]. Thus, eggs from strains in temperate regions are moderately tolerant to cold and can even tolerate short durations of freezing temperatures [34], [35] . Female *Ae. albopictus* lay eggs in human-made and natural containers just above the waterline. Reported flight range of this species is typically less than 200 m [36].

Several laboratory studies on *Ae. albopictus* vector competence for the CHIKV LR 226OPY1 epidemic strain have now been conducted reporting a range of dissemination rates from 26–100% in various geographic strains of the vector [20], [37]–[40]. A recent laboratory study, using salivary gland infection as a proxy for transmission, demonstrated transmission rates from above 67% for Galveston, TX strain, Florida strain, and a New Jersey/New York metropolitan strain (Harrington, Sanchez-Vargas and Olson, unpublished data).

Concerns for the role of *Ae. albopictus* as an active disease vector have been raised since its introduction and in the USA [41], [42]. Since introduction, *Ae. albopictus* has become established in 26 states primarily in Southeast, gulf coast and mid-Atlantic regions. The species is currently expanding its range through New Jersey and into New York State [43], [44]. Given the establishment of *Ae. albopictus* in these regions, travel related introductions of several arboviruses suggest a potential increase in epidemic risk for the USA. High numbers of CHIKF cases are periodically reported in US travelers [45], [46]. However, local CHIKV outbreaks have not been detected in the US to date, presumably because of the asynchrony between the arrival of the exposed individuals and the abundance of the vectors [45].

In this study, we explicitly evaluated the risk of epidemic events by simulating the introduction of Chikungunya virus into three naïve US populations. Assuming established mosquito populations in each area, we introduced one exposed individual to evaluate the epidemic potential size of an outbreak, taking into account the population dynamics of the vector and its susceptibility to temperature regimes. We predicted low epidemic risk for disease introduction during periods of low vector abundance and high epidemic risk for certain critical periods that show increasing, or high, vector abundance. These results provide valuable additional information not only for early warning systems but also for the implementation of intervention strategies with the goal of reducing vector populations or human risk of exposure.

## Methods

**Figure 1:**
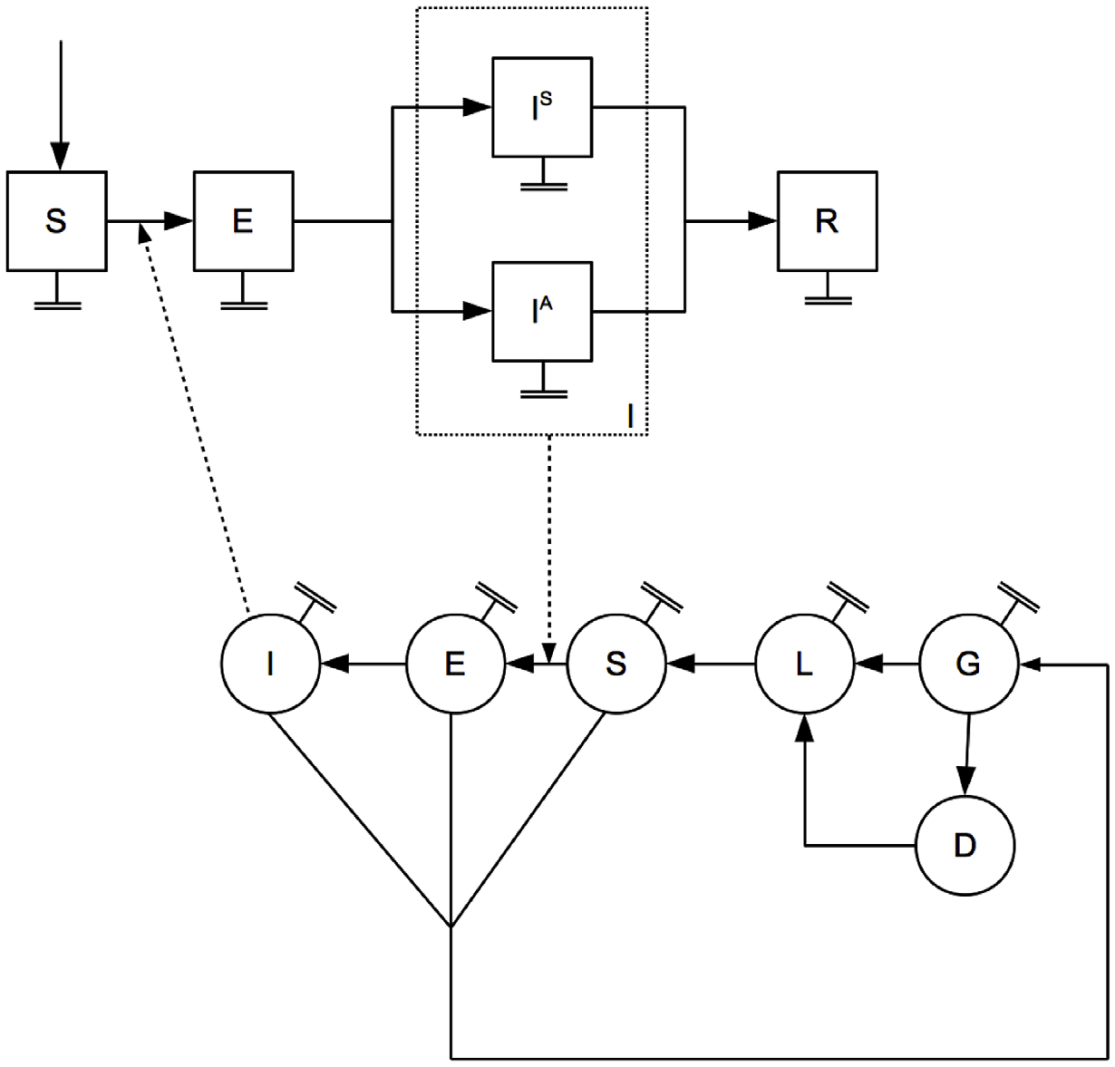
Graphical representation of the model. Squares (circles) represent the dynamic model for humans (mosquitoes). Human population is divided into susceptible (S), exposed (E), symptomatic (I^S^) and asymptomatic (I^A^) infective, and recovered (R) individuals. Mosquito population is divided into immature eggs (G), larvae (L) and eggs under a diapause (D) state, and mature susceptible (S), exposed (E) and infected (I) states. Full arrows represent transition from one state to the other. Lines with parallel end represent natural mortality. Dotted lines represent infection dynamics. A full description of the parameters and the model can be found in Material S1.

**Figure 2:**
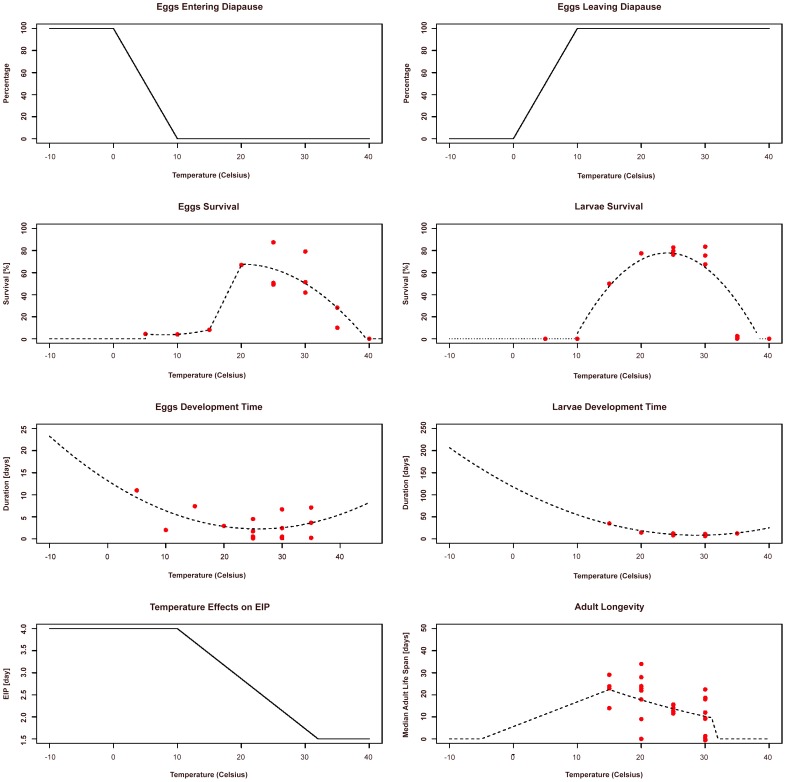
Variation of parameter values with temperature. Panels with full lines represent functional shapes based on assumptions, those with dotted lines are functional shapes fitted to data (points). See Material S1 for a full mathematical representation of these functions.

To study the dynamics of the introduction of CHIKV in an immunologically naive population we constructed a model with demographic stochasticity for mosquitoes and humans (Figure 1, see Material-S1 for model equations). Using a classical approach, the human host population was divided into susceptible (S), exposed (E), symptomatic infective (I^S^), asymptomatic infective (I^A^) and recovered (R) classes. Analogously, the adult mosquito population was divided into susceptible (S), exposed (E) and infected (I) classes. In addition, we considered the immature stages of mosquito population, including mosquito eggs (G), larvae and pupae (L) and eggs undergoing diapause (D). Vital mosquito rates in this model were temperature dependent (Table S1 and Figure 2). Density-dependent effects were added to both mosquito and human populations. It is worthwhile to note that although the net effect of having density-dependent terms in the model is to avoid uncontrolled population growth, they represent a broad range of factors from larval overcrowding effects to human behavior.

The functional forms of the temperature forcing on the parameters for the dynamic of the vector population are presented in Figure 2 (The mathematical forms are presented in Material S1). Two temperature thresholds (T_sD_ and T_eD_) were used to determine the diapause state. Eggs entered diapause (i.e., arrested development) when temperature was below T_sD_, and eggs do not undergo diapause for temperatures above T_eD_. The proportion of eggs undergoing (avoiding) the diapause state linearly decreased (increased) with increasing temperature for environmental conditions between T_sD_ and T_eD_. Although the determination of diapause periods follows a complex combination of factors — including temperature and photoperiod— temperature was used as a proxy for such combination in here. Dependence on temperature of both egg survival and development time was fitted to experimental data (Harrington, unpublished data) and reports from the literature [47], [48]. Similarly, experimental and reported data were used for the fitting of temperature influence on larvae survival and development time [Harrington, unpublished data, [47], [48]. Adult longevity dependence on temperature was fitted to experimental data using the general assumption that longevity declines linearly with temperatures under 10°C [49]. Although there is no experimental support reported in the literature, CHIKV extrinsic incubation period (EIP) was assumed to be reduced with increasing temperature (up to 32°C) as with other arboviruses such as DENV [50]. The reported minimum extrinsic incubation period for CHIKV in several studies is 2 days [15]. Hence, we modeled EIP as a linear function with a minimum duration of 1.5 days at 32°C and a maximum duration of 4 days at 10°C [15], [51]. For the purposes of the current model we assumed transmission rates based on early experimental work [52].

Daily temperatures were calculated by applying a spline interpolation to the monthly mean temperature data of the last decade obtained from the Intergovernmental Panel on Climate Change (http://www.ipcc-data.org) (Figure S1). The model was run using temperature data from different locations to evaluate variability on epidemic risk with temperature patterns. Here, we present the results for three major US ports of entry that encompass a wide seasonal variation in temperature: New York, Atlanta, and Miami.

Population sizes and carrying capacity parameters for human populations were estimated using the city size data reported in the last census (http://www.census.gov). Mosquito population carrying capacity was estimated assuming a maximum number of vectors per host. We ran independent simulations for the three ports of entry changing this ratio (we present here the results for 0.5, 1 and 3 mosquitoes per human).

**Figure 3:**
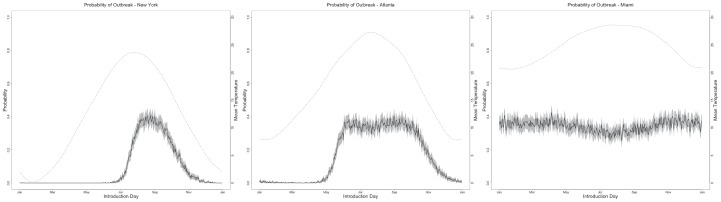
Probability of Outbreak. Probability of outbreak (left y-axes) as a function of day of introduction of CHIKV (x-axes) with a ratio of vector to hosts equals to 0.5 and 100% for meal preference. Full dark lines represent the mean of 1000 Monte Carlo simulations. Gray areas represent the standard deviation. Dotted lines are temperature values for the different locations (right y-axes). See Material S1 for other parameter combinations.

**Figure 4:**
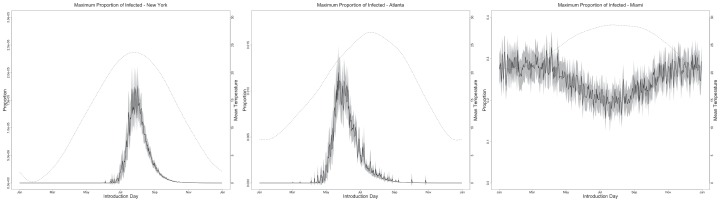
Proportion of infected individuals. Proportion of infected individuals (left y-axes) as a function of day of introduction of CHIKV (x-axes) with a ratio of vector to host equals to 0.5 and 100% for meal preference. Notice different panels have different left y-axes limits. Full dark lines represent the mean of 1000 Monte Carlo simulations. Gray areas represent the standard deviation. Dotted lines are temperature values for the different locations (right y-axes). See Material S1 for other parameter combinations.

The model was run for five years. Initial population sizes in the model were selected according to the expected equilibrium values. During the first year of simulation there was no disease present in the model and therefore both human and mosquito populations drifted to their respective equilibria. CHIKV was introduced into the model during the second year of simulation via one exposed individual, and the simulation was run until the end of the fifth year. We calculated the final number of infective individuals, the number of infected at the outbreak peak, and the time to reach the outbreak peak from the day of introduction for each one of 1000 Monte Carlo simulations. We ran the simulations systematically varying the day of introduction of the disease from January 1st to December 31st, which allowed us to express the outbreak probability as a function of the introduction day (Figure 3). Here, the probability of outbreak was defined as the frequency of cases where the chain of infection was functional (i.e., the number of infected individuals was bigger than 1). In addition, we calculated the risk of an outbreak as the mean of the final epidemic size (summation of all infected individuals) over the average susceptible population size (Figure 4). These simulations were replicated not only varying the ratio of mosquitoes to humans (considering values of 0.5, 1 and 3 for that ratio) but also reducing the mosquito feeding pattern for human blood from 100% to 25%.

## Results

Figure 3 displays the probability of outbreak for the different locations as function of the day of introduction and 0.5 mosquitoes to human ratio for each location, and a 100% human meal preference (See Material S1 for results with other vector to host ratios and human blood meal patterns). The probability of an outbreak (defined as at least one successful transmission event to a human) for New York shows a peak around 38% for a CHIKV introduction in August, and is over 30% during the interval from August 6^th^ to September 11^th^. In addition, there is a significant probability of outbreak after an introduction on June 15^th^ and up to December. The probability of having an outbreak late in November is very small and it is a consequence of using mean monthly temperature data as a basis for the temperature patterns.

Outbreaks also were seasonal for Atlanta, with no significant probability of outbreaks after introductions between January 12^th^ and April 9^th^. Moreover, in Atlanta, the probability of outbreak was greater than 30% for a longer period, extending from June 6^th^ to September 26^th^, with peak values similar to those in New York. In contrast, for Miami chances of a CHIKV outbreak were significant after an introduction at any time during the year.

Our model only demonstrated the occurrence of at least one successful transmission event, however, the maximum prevalence reached for those outbreaks is likely to be a more important parameter (Figure 4). Consequently, we explored the peak infection rate with our model and found that it varies significantly between locations and also with the ratio of vectors to hosts and human feeding patterns (see Tables S2 and S3 for results with other vector to host ratios and meal preferences). When we set the ratio of mosquitoes to one human at 0.5 and human feeding rates at 100%, peak infection in New York was very small (0.0002%), peaking at the beginning of the high probability outbreak period. A similar pattern, but with higher prevalence values for CHIK in humans (0.1381%) was observed for Atlanta. In Miami, however a high mean prevalence for CHIK in humans (25.0187%) was observed throughout the year.

Additionally, we calculated the number of days from pathogen introduction until the peak prevalence (Table 1). These calculations reveal that (in general), when mosquito feeding preference is set to be only from humans, the time to peak prevalence is longer than when feeding preferences are broad. Thus, when human blood feeding is low, the epidemic peak usually occurs shortly after introduction because the chain of transmission could be easily interrupted. However, when human blood feeding is set to 100%, the epidemic peaks occurs within 20 days for cities in cooler climates (i.e. New York) and approximately one to three months for warmer locations. In these warmer locations several secondary cases are expected to follow the index case, and the stochastic interruptions of the chain of infection can only slow the development of the epidemic instead of stopping it.

**Table 1:** Mean (and interquartile distance) for the number of days from the pathogen introduction to the epidemic peak.

	Meal Preference		
	Vector/Host Ratio	25%	100%
New York	0.5	4.960 (4.62–5.06)	9.759 (4.64–10.11)
	1	5.277 (4.61–5.39)	14.732 (4.66–16.71)
	3	7.080 (4.63–7.43)	23.514 (4.70–35.62)
Atlanta	0.5	5.146 (4.67–5.55)	21.182 (4.89–37.32)
	1	5.772 (4.73–6.84)	45.704 (5.11–80.92)
	3	10.458 (4.84–17.32)	55.390 (5.92–74.56)
Miami	0.5	5.655 (5.35–5.93)	87.719 (77.34–97.48)
	1	7.006 (6.43–7.51)	88.723 (84.77–92.34)
	3	35.520 (26.87–42.72)	73.876 (72.66–75.24)

Average (first – third quartile) number of days from the introduction of an exposed individual to the epidemic peak at different locations, ratios of mosquitoes to humans, and meal preference.

## Discussion

Since the occurrence of the CHIKF outbreak in Italy in 2007, the risk of similar outbreaks in the United States and other temperate countries has become a public health concern [42]. Our models, based on the introduction of one exposed individual, show that the probability of an outbreak in any of the three chosen locations varies by geographic location. As expected, for areas like the New York metropolitan region and Atlanta, that display a strong seasonality in temperature and therefore in mosquito abundance, this risk is bounded to the summer time and low prevalence levels are predicted. In locations with temperature patterns similar to those in Miami, which allow for year-round mosquito activity, the risk for outbreak is not bounded seasonally. In addition, predicted prevalence levels are higher for Miami because usually the chain of infection is not completely interrupted. These models also show that the proportion of people affected in an outbreak is reduced dramatically with increasing latitude (Figure 4). Furthermore, the models suggest the replenishment of susceptible individuals is not enough to create endemic foci, however this result could change when using more realistic models.

Our model outputs display higher sensitivity to parameters controlling the proportion of blood meals from humans than the vector: host ratio. Nevertheless, the increased ratio of mosquitoes to humans led to a two-fold increase in the probability of outbreak at all locations. This result highlights the importance of vector control to reduce both the risk of outbreaks and the proportion of infected individuals. It is important to note that, in this modeling approach, both parameters may be interpreted as proxies for a reduction of human exposure to mosquitoes. Hence, our results confirm the relevance of public campaigns advising residents to control mosquitoes at home and take precautions to avoid mosquito exposure to reduce disease outbreaks.

The time between CHIKV introduction and peak outbreaks revealed that, for locations with temperature patterns similar to those of Miami where mosquito populations may not undergo diapause, CHIKV infections might circulate at low levels for several months until reaching dramatic proportions. Early detection of cases in these regions will be important to reduce the magnitude of an outbreak. However, in locations such as New York and Atlanta, a critical temporal window for interventions could be identified and intervention during such periods may be enough to significantly reduce the probability of an outbreak.

It is clear that these predictive models are highly sensitive to temperature patterns that govern mosquito population dynamics, and could be improved by using non-averaged temperature data (i.e. sampling from the distribution of temperatures), and including other environmental factors such as rainfall and photoperiod that can have a significant influence on vector populations. In addition, reduction of individual exposure (only modeled as a reduction in human feeding patterns here) should be considered in order to have more accurate predictions. This modeling approach highlights the fact that a better understanding of epidemiological dynamics will require further studies on both biological and non-biological processes. Especially important will be: (1) further studies on diapause, abundance and feeding biology of *Ae. albopictus*, (2) the inclusion of multiple disease introduction events, either simultaneously of temporally spread, and (3) a better understanding of the evolution and plasticity of both pathogen and vector.

Our results strongly suggest that, in the event of an introduction and establishment of CHIKV in the United States, endemic and epidemic regions would emerge initially, mainly defined by environmental factors controlling annual mosquito population cycles. These regions should be identified in order to plan different intervention measures. In addition, reducing mosquito population sizes (and, consequently, reducing vector: human ratios) can lower the probability and magnitude of outbreaks mainly for regions with strongly marked seasonal temperature patterns.

Typical control strategies for vector borne diseases are: (1) reduction of vector population, (2) reduction of host exposure to infectious mosquito bites, and (3) isolation of infective hosts. This model also allows for evaluation of the effects of changes in the mosquito feeding patterns. Simulation results suggest that a reduction of vector population and human exposure could be very effective for a reduction of both the risk of an outbreak and the population at risk.

The results presented here simulating significant CHIK outbreaks in the US were based on a conservative approach of one exposed individual introduced to a region [45]. Given CHIKV infections in returning US travelers [45], [46] and the low numbers of infected individuals needed to spark an outbreak, we conclude that US health systems should be vigilant.

## Supporting Information

Table S1 **Parameters and functions of the model** Parameter values definition and functions of the model. Mathematical forms for the temperature dependent parameters are presented in Material S1.
(DOC)

Table S2 **Mean (maximum) probability of outbreak** Mean (maximum) probability of outbreak for different locations, ratio of mosquitoes to humans, and meal preferences. The table shows an increase in the probability of an outbreak either by increasing the ratio of vectors to hosts or by increasing the host meal preference.
(DOC)

Table S3 **Proportion of infected (maximum)** Mean (maximum) proportion of infected individual for different locations, ratio of mosquitoes to humans, and meal preferences. The table shows an increase in the probability of an outbreak either by increasing the ratio of vectors to hosts or by increasing the meal preference.
(DOC)

Figure S1 **Temperature patterns for the simulated locations.** Full line corresponds to Miami, dashed line to Atlanta and dotted line to New York. Data were calculated based on the last decade monthly temperature and applying a spline interpolation for the daily
(DOC)

Material S1 (DOC)
