# Supplementary material for: Modeling Dynamic Introduction of Chikungunya Virus in the United States
Source: PLoS Negl Trop Dis. 2012 Nov 29;6(11):e1918. doi: 10.1371/journal.pntd.0001918 (PMC3510155; doi:10.1371/journal.pntd.0001918)
Supplement: Table S1 — Parameters and functions of the model Parameter values definition and functions of the model. Mathematical forms for the temperature dependent parameters are presented in Material S1. (DOC) [file pntd.0001918.s001.doc]

SUPPLEMENTARY INFORMATION FOR **MODELING DYNAMIC INTRODUCTION OF CHIKUNGUNYA VIRUS IN THE UNITED STATES** Ruiz-Moreno D, Sanchez Vargas I, Olson KE and Harrington, LC

### **Table S1: Parameters and functions of the model**

| **Parameter** | **Description** | **Value** |
| --- | --- | --- |
| $\rho_{H}$ | Strength of density dependence for human population | 5.514e-05 (Miami)  1.901e-05 (Atlanta)  1.225e-06 (New York) |
| $\mu_{S}$, $\mu_{E}$, $\mu_{I^{S}}$, $\mu_{I^{A}}$, $\mu_{R}$ | Human mortality rate | 3.425e-05 assuming a mean longevity of 80 years |
| $\eta_{I^{S}}$, $\eta_{I^{A}}$ | Disease induced mortality | 1/1000 [52] |
| $\frac{1}{\gamma_{I^{S}}}$, $\frac{1}{\gamma_{I^{A}}}$ | Intrinsic incubation period | 4 [1, 8-11] |
| $\frac{1}{\gamma}$ | Infective period | 0.158 [1] |
| c | Symptomatic/Asymptomatic | 0.75 [3] |
| b | Mosquito biting rate | 1/3 |
| T_HM_ | Probability of infection (from mosquitoes to humans) | 0.726 [52] |
| T_MH_ | Probability of infection (from humans to mosquitoes) | 0.977 [52] |
| $f$ | Average number of eggs per mosquito per day | 4.4 [54-57] |
| $\rho_{M}$ | Strength of density dependence for mosquito population | 6.029e-06 (Miami – ratio 0.5)  3.029e-06 (Miami – ratio 1)  1.000e-06 (Miami – ratio 3)  5.029e-06 (Atlanta – ratio 0.5)  2.529e-06 (Atlanta – ratio 1)  8.295e-07 (Atlanta – ratio 3)  2.800e-07 (New York – ratio 0.5)  1.400e-07 (New York – ratio 1)  4.705e-08 (New York – ratio 3) |
| $\mu_{A_{S}}$,$\mu_{A_{E}}$,$\mu_{A_{I}}$ | Adult mosquito mortality | Temperature Dependent (Fig. 2) |
| $\mu_{E}$ | Mosquito egg mortality | Temperature Dependent (Fig. 2) |
| $\xi_{E}$ | Mosquito egg maturation rate | Temperature Dependent (Fig. 2) |
| $\xi_{L}$ | Mosquito larval maturation rate | Temperature Dependent (Fig. 2) |
| $\mu_{L}$ | Mosquito larval mortality | Temperature Dependent (Fig. 2) |
| $\frac{1}{\sigma_{A_{E}}}$ | Extrinsic incubation period | Temperature Dependent (Fig. 2) |
| $\zeta_{G}$ | Fraction of mosquito eggs entering diapause | Temperature Dependent (Fig. 2) |
| $\nu_{D}$ | Fraction of mosquito eggs leaving diapause | Temperature Dependent (Fig. 2) |
| $b\cdot T_{MH}\cdot\frac{I_{M}}{N_{H}}$ | Force of infection term for humans |  |
| $b\cdot T_{HM}\cdot\frac{I^{S}+I^{A}}{N_{H}}$ | Force of infection term for mosquitoes |  |

Parameter values definition and functions of the model. Mathematical forms for the temperature dependent parameters are presented in the supplementary material.
