# Supplementary material for: Modeling Dynamic Introduction of Chikungunya Virus in the United States
Source: PLoS Negl Trop Dis. 2012 Nov 29;6(11):e1918. doi: 10.1371/journal.pntd.0001918 (PMC3510155; doi:10.1371/journal.pntd.0001918)
Supplement: Table S2 — Mean (maximum) probability of outbreak Mean (maximum) probability of outbreak for different locations, ratio of mosquitoes to humans, and meal preferences. The table shows an increase in the probability of an outbreak either by increasing the ratio of vectors to hosts or by increasing the host meal preference. (DOC) [file pntd.0001918.s002.doc]

SUPPLEMENTARY INFORMATION FOR **MODELING DYNAMIC INTRODUCTION OF CHIKUNGUNYA VIRUS IN THE UNITED STATES** Ruiz-Moreno D, Sanchez Vargas I, Olson KE and Harrington, LC

### **Table S2: Mean (maximum) probability of outbreak**

|  |  | Meal Preference | |
| --- | --- | --- | --- |
|  | Vector/Host Ratio | 25% | 100% |
| New York | 0.5 | 0.0097 (0.074) | 0.0868 (0.408) |
|  | 1 | 0.0196 (0.134) | 0.1373 (0.61) |
|  | 3 | 0.0582 (0.336) | 0.2271 (0.83) |
| Atlanta | 0.5 | 0.0173 (0.066) | 0.1651 (0.408) |
|  | 1 | 0.0359 (0.12) | 0.2592 (0.596) |
|  | 3 | 0.1116 (0.322) | 0.4162 (0.836) |
| Miami | 0.5 | 0.0367 (0.062) | 0.3441 (0.426) |
|  | 1 | 0.0766 (0.12) | 0.5249 (0.602) |
|  | 3 | 0.2366 (0.312) | 0.7879 (0.862) |

Mean (maximum) probability of outbreak for different locations, ratio of mosquitoes to humans, and meal preferences. The table shows an increase in the probability of an outbreak either by increasing the ratio of vectors to hosts or by increasing the host meal preference.
