# Supplementary material for: Modeling Dynamic Introduction of Chikungunya Virus in the United States
Source: PLoS Negl Trop Dis. 2012 Nov 29;6(11):e1918. doi: 10.1371/journal.pntd.0001918 (PMC3510155; doi:10.1371/journal.pntd.0001918)
Supplement: Table S3 — Proportion of infected (maximum) Mean (maximum) proportion of infected individual for different locations, ratio of mosquitoes to humans, and meal preferences. The table shows an increase in the probability of an outbreak either by increasing the ratio of vectors to hosts or by increasing the meal preference. (DOC) [file pntd.0001918.s003.doc]

SUPPLEMENTARY INFORMATION FOR **MODELING DYNAMIC INTRODUCTION OF CHIKUNGUNYA VIRUS IN THE UNITED STATES** Ruiz-Moreno D, Sanchez Vargas I, Olson KE and Harrington, LC

### **Table S3: Proportion of infected (maximum)**

|  |  | Meal Preference | |
| --- | --- | --- | --- |
|  | Vector/Host Ratio | 25% | 100% |
| New York | 0.5 | 7.08e-09 (4.65e-08) | 1.66e-06 (1.76e-05) |
|  | 1 | 1.65e-08 (1.17e-07) | 5.06e-05 (6.36e-05) |
|  | 3 | 9.57e-08 (7.23e-07) | 3.34e-02 (4.18e-01) |
| Atlanta | 0.5 | 2.11e-07 (7.44e-07) | 1.38e-02 (1.20e-02) |
|  | 1 | 5.16e-07 (1.85e-06) | 9.22e-02 (4.62e-01) |
|  | 3 | 5.05e-06 (2.23e-05) | 3.17e-01 (8.34e-01) |
| Miami | 0.5 | 5.23e-07 (1.08e-06) | 2.50e-01 (3.27e-01) |
|  | 1 | 1.31e-06 (2.47e-06) | 4.99e-01 (5.72e-01) |
|  | 3 | 1.46e-05 (4.44e-05) | 7.87e-01 (8.60e-01) |

Mean (maximum) proportion of infected individual for different locations, ratio of mosquitoes to humans, and meal preferences. The table shows an increase in the probability of an outbreak either by increasing the ratio of vectors to hosts or by increasing the meal preference.
