# Supplementary material for: Modeling Dynamic Introduction of Chikungunya Virus in the United States
Source: PLoS Negl Trop Dis. 2012 Nov 29;6(11):e1918. doi: 10.1371/journal.pntd.0001918 (PMC3510155; doi:10.1371/journal.pntd.0001918)
Supplement: Figure S1 — Temperature patterns for the simulated locations. Full line corresponds to Miami, dashed line to Atlanta and dotted line to New York. Data were calculated based on the last decade monthly temperature and applying a spline interpolation for the daily (DOC) [file pntd.0001918.s004.doc]

SUPPLEMENTARY INFORMATION FOR **MODELING DYNAMIC INTRODUCTION OF CHIKUNGUNYA VIRUS IN THE UNITED STATES** Ruiz-Moreno D, Sanchez Vargas I, Olson KE and Harrington, LC


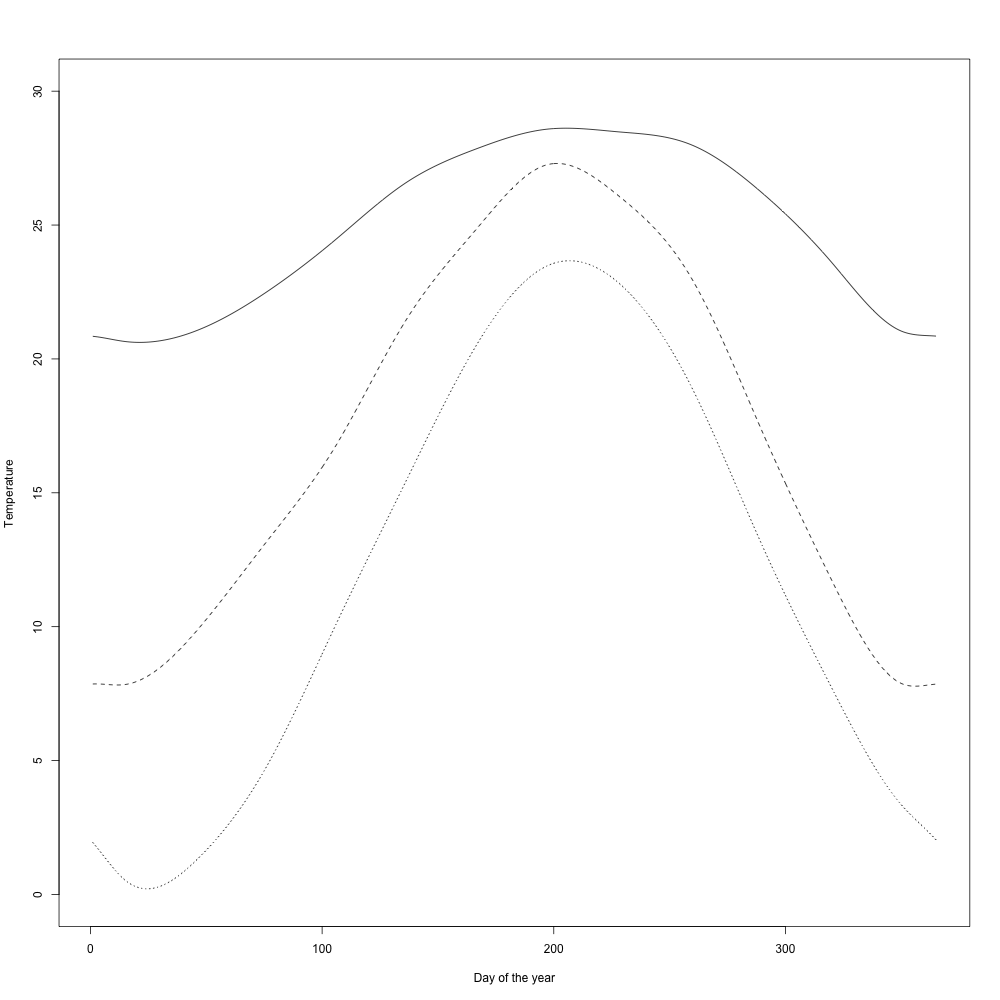
**Figure S1: Temperature patterns for the simulated locations.** Full line corresponds to Miami, dashed line to Atlanta and dotted line to New York. Data were calculated based on the last decade monthly temperature and applying a spline interpolation for the daily values.
