# Supplementary material for: Modeling Dynamic Introduction of Chikungunya Virus in the United States
Source: PLoS Negl Trop Dis. 2012 Nov 29;6(11):e1918. doi: 10.1371/journal.pntd.0001918 (PMC3510155; doi:10.1371/journal.pntd.0001918)
Supplement: Material S1 — (DOC) [file pntd.0001918.s005.doc]

SUPPLEMENTARY INFORMATION FOR **MODELING DYNAMIC INTRODUCTION OF CHIKUNGUNYA VIRUS IN THE UNITED STATES** Ruiz-Moreno D, Sanchez Vargas I, Olson KE and Harrington, LC

### **MODEL EQUATIONS AND DETAILS**

We used an ordinary differential equation model for CHIKF. This model included demographic population dynamics for both mosquitoes and humans. For the mosquito component we divided the population into eggs (G), eggs undergoing diapause (D), larvae (L) and adults that could be susceptible (A_S_), exposed (A_E_) or infected (A_I_).

$$\frac{dG}{dt}=f\cdot A_{M}\cdot e^{-\rho_{M}\cdot A_{M}}-\mu_{G}\cdot G-\zeta_{G}\cdot G-\xi_{G}\cdot G$$

$$\frac{dD}{dt}=\zeta_{G}\cdot G-\nu_{D}\cdot D$$

$$\frac{dL}{dt}=\xi_{G}\cdot G+\nu_{D}\cdot D-\mu_{L}\cdot L-\xi_{L}\cdot L$$

$$\frac{dA_{S}}{dt}=\xi_{L}\cdot L\cdot e^{-\rho_{A}\cdot A_{M}}-\mu_{A_{S}}\cdot A_{S}-\frac{b}{N_{H}}\cdot T_{MH}\cdot\left(I^{S}+I^{A}\right)\cdot A_{S}$$

$$\frac{dA_{E}}{dt}=\frac{b}{N_{H}}\cdot T_{MH}\cdot\left(I^{S}+I^{A}\right)\cdot A_{S}-\mu_{A_{E}}\cdot A_{E}-\sigma_{A_{E}}\cdot A_{E}$$

$$\frac{dA_{I}}{dt}=\sigma_{A_{E}}\cdot A_{E}-\mu_{A_{I}}\cdot A_{I}$$

In the model $A_{M}$ represents the total population of adult mosquitoes, $N_{H}$is the total population size for humans and $I_{S}$ and $I_{A}$ the symptomatic and asymptomatic infected humans. Mosquito population growth was controlled by density dependent factors for reproduction $\rho_{M}$ and maturation into adulthood $\rho_{A}$ (both parameters were set to the same value). Parameters $\mu_{G}$, $\zeta_{G}$, $\xi_{G}$, represent the natural mortality of eggs, transition to the diapause and transition to the larval states respectively. The parameter $\nu_{D}$defines the transition from eggs undergoing diapause into the larval stage. $\mu_{L}$ and $\xi_{L}$ respectively represent the mortality and maturation of larvae, $\mu_{A_{S}}$, $\mu_{A_{E}}$ , and $\mu_{A_{I}}$ are the mortality of adult mosquito individuals and $\sigma_{A_{E}}$represents the extrinsic infection period. Disease transmission was modeled using the biting rate ($b$) and the effective virus transmission from mosquitoes to humans $T_{MH}$ (and from human to mosquitoes, $T_{HM}$, on the human side).

Several mosquito population parameters ($\mu_{G}$, $\zeta_{G}$, $\xi_{G}$, $\nu_{D}$, $\mu_{L}$, $\xi_{L}$, $\mu_{A_{S}}$, $\mu_{A_{E}}$, $\sigma_{A_{E}}$, $\mu_{A_{I}}$ ) were temperature dependent (Figure 2 and analytical formulations below).

The human population was divided into susceptible ($S$), exposed ($E$), symptomatic ($I^{S}$) and asymptomatic ($I^{A}$) infected, and recovered ($R$) individuals.

$$\frac{dS}{dt}=N_{H}\cdot e^{-\rho_{H}\cdot N_{H}}-\mu_{S}\cdot S-\frac{b}{N_{H}}\cdot T_{HM}\cdot A_{I}\cdot S$$

$$\frac{dE}{dt}=\frac{b}{N_{H}}\cdot T_{HM}\cdot A_{I}\cdot S-\sigma_{E}\cdot E-\mu_{E}\cdot E$$

$$\frac{dI^{S}}{dt}=c\cdot\sigma_{E}\cdot E-\gamma_{I^{S}}\cdot I^{S}-\mu_{I^{S}}\cdot I^{S}-\eta_{I^{S}}\cdot I^{S}$$

$$\frac{dI^{A}}{dt}=\left(1-c\right)\cdot\sigma_{E}\cdot E-\gamma_{I^{A}}\cdot I^{A}-\mu_{I^{A}}\cdot I^{A}-\eta_{I^{A}}\cdot I^{A}$$

$$\frac{dR}{dt}=\gamma_{I^{A}}\cdot I^{A}+\gamma_{I^{S}}\cdot I^{S}-\mu_{R}\cdot R$$

Natural mortality for the corresponding classes of individuals is represented by $\mu$. The intrinsic infection period is modeled by $\sigma_{E}$and disease induced mortality by $\eta$. The constant c represents the proportion of symptomatic individuals, and $\gamma$ is the inverse of the infectious period.

The model was simulated using discrete population sizes and an adaptive tau-leap method for simulating differential equations (see Keeling and Rohani, 2008 for implementation details). The algorithm runs with $\delta t=\frac{1}{10}$ day, unless a high number of events that lead to negative population sizes occurs. In such cases, the value of $\delta t$ is reduced by half as many time as necessary to avoid negative values in population sizes.

### **TEMPERATURE DEPENDENT FUNCTIONS**

Mosquito Egg Survival

$$f(temp)=\left\{\begin{matrix} 0 & temp\leq5 \\ 9.4-1.46\cdot temp+0.092\cdot temp^{2} & 5<temp\leq15 \\ -167.9+11.74\cdot temp & 15<temp\leq20 \\ -5.08+7.23\cdot temp-0.18\cdot temp^{2} & 20<temp\leq39.54 \\ 0 & 39.54<temp \end{matrix}\right.$$

Proportion of eggs entering diapause state

$$f(temp)=\left\{\begin{matrix} 0 & temp<0 \\ -0.1\cdot temp+1 & 0\leq temp\leq10 \\ 1 & 10<temp \end{matrix}\right.$$

Egg maturation period

$$f\left(temp\right)=13.18-0.845\cdot temp+0.016\cdot temp^{2}$$

Proportion of eggs leaving diapause state

$$f(temp)=\left\{\begin{matrix} 0 & temp<0 \\ 0.1\cdot temp & 0\leq temp\leq10 \\ 1 & 10<temp \end{matrix}\right.$$

Survival of Larvae

$$f(temp)=\left\{\begin{matrix} 0 & temp<10 \\ -136.08+17.8\cdot temp-0.37\cdot temp^{2} & 10\leq temp\leq38.5 \\ 0 & 38.5<temp \end{matrix}\right.$$

Larvae Maturation

$$f\left(temp\right)=117.58-7.6\cdot temp+0.1321528\cdot temp^{2}$$

Adult Survival

$$f(temp)=\left\{\begin{matrix} 0 & temp<-5 \\ 5.6+1.12\cdot temp & -5\leq temp<15 \\ 40.27-1.38\cdot temp+0.01\cdot temp^{2} & 15\leq temp\leq38.5 \\ 0 & 38.5<temp \end{matrix}\right.$$

Extrinsic Incubation Period

$$f(temp)=\left\{\begin{matrix} 4 & temp<10 \\ \frac{113}{22}-\frac{2.4}{22}\cdot temp & 10\leq temp\leq32 \\ 1.5 & 32<temp \end{matrix}\right.$$
